# Pre-diagnostic intake of vitamin D and incidence of colorectal cancer by anatomical subsites: the Norwegian Women and Cancer Cohort Study (NOWAC)

**DOI:** 10.1017/S0007114523000077

**Published:** 2023-09-28

**Authors:** Elise Marlen Paulsen, Charlotta Rylander, Magritt Brustad, Torill E Jensen

**Affiliations:** Department of Community Medicine, University of Tromsø – The Arctic University of Norway, 9037 Tromsø, Norway

**Keywords:** Vitamin D intake, Colorectal cancer, Colorectal cancer by anatomical subsites, Incidence, Prospective

## Abstract

According to the World Cancer Research Fund International, vitamin D might decrease the risk of colorectal cancer (CRC). However, less is known about the association with cancers in different subsites of the colon and in the rectum. The aim of this study was to examine associations between pre-diagnostic intake of vitamin D and risk of CRC by anatomical subsites. Data from 95 416 participants in the Norwegian Women and Cancer Cohort Study was included, and vitamin D intake was estimated from two repeated FFQ. Associations between vitamin D intake and incidence of CRC were assessed using multivariable Cox regression. During follow-up, there were 1774 incident cases of CRC. A small but borderline significant inverse association was found for a 5-µg increase in vitamin D intake and risk of CRC (hazard ratio (HR) = 0·97; 95 % CI 0·93, 1·01) and colon cancer (HR = 0·96; 95 % CI 0·91, 1·01). High (≥ 20 µg) compared with low (< 10 µg) vitamin D intake was associated with 17 % borderline significant reduced risk of CRC (HR = 0·83; 95 % CI 0·68, 1·02). Medium (10–19 µg) *v*. low intake (< 10 µg) was associated with 27 % reduced risk of proximal colon cancer (HR = 0·73; 95 % CI 0·57, 0·94). No significant associations were observed between vitamin D intake and risk of distal colon or rectal cancer. Our study indicates that vitamin D may be differently associated with subsites of the colon. The association between vitamin D intake and proximal colon cancer is novel.

Worldwide, colorectal cancer (CRC) is the third most common cancer among men and the second most common among women^(1)^. In Norway, CRC is the second most common form of cancer among both men and women, and the number of cases has more than doubled in the last half-century^(2)^. In fact, it is believed that within 8 to 12 years, there will be more than 6000 incident cases yearly, an increase of more than 40 % since 2016^(2)^. The CRC incidence in Norway is higher than in the other Nordic countries, without any current explanation^(2)^. Nonetheless, it is believed that a large proportion of CRC cases can be prevented through a healthy diet and lifestyle^(3)^. According to the World Cancer Research Fund (WCRF), there is strong evidence that consumption of wholegrains, foods containing dietary fibre and dairy products decreases the risk of CRC^(3)^. Furthermore, being physically active is associated with decreased risk of colon cancer^(3)^. In contrast, there is strong evidence that consumption of red and processed meat, alcoholic drinks, being overweight or obese, and being tall is associated with increased risk of CRC^(3)^.

Vitamin D is a fat-soluble vitamin that can be formed endogenously in the skin by exposure to sunlight or obtained from certain sources in the diet. Fatty fish, liquid cod liver oil, edible fats and milk products are the major dietary sources^(4)^. In 1980, Garland et al.^(5)^ hypothesised that the higher prevalence of colon cancer in certain geographical areas could be explained by less exposure to sunlight and consequently, low vitamin D status. Since then, several studies have been published investigating associations between vitamin D and CRC, either by estimating intake of vitamin D through foods and/or supplements or by measuring 25-hydroxyvitamin D (25(OH)D) in serum, but the results are still inconsistent^(6–15)^. A meta-analysis published in 2011 conducted by the WCRF supported an association between intake of vitamin D, serum 25(OH)D, the BsmI vitamin D receptor polymorphism and CRC, but concluded a need for supportive evidence^(16)^. The conclusion in the third WCRF Continuous Update Project report published in 2018 has not changed due to lack of sufficient scientific evidence^(3)^. Nevertheless, a review of meta analyses from 2021^(17)^ was generally consistent with the findings by WCRF. WCRF has also pointed out that cancer in different subsites of the colon and rectum may have different pathogenesis and different causal factors^(3)^.

Few studies have examined associations between intake of vitamin D and risk of cancer in different subsites of the colon and rectum^(6,8–10,12)^. Therefore, we aimed to investigate pre-diagnostic intake of vitamin D and the incidence of cancer in different anatomical subsites of the colon and rectum among Norwegian women. Specifically, we studied whether an increase in vitamin D intake or an intake in line with the Nordic Nutrition Recommendations for vitamin D^(4)^ was associated with CRC, colon cancer, proximal colon cancer, and distal colon and rectal cancer.

## Methods

### Study design and study sample

The Norwegian Women and Cancer Cohort Study (NOWAC) is a large prospective cohort that was initiated in 1991^(18)^. In brief, a random sample of women was selected from the National Registry in Norway, and approximately 172 000 were enrolled in NOWAC between 1991 and 2007^(19)^. The participants completed self-administered questionnaires, including questions about reproductive factors, anthropometrics, demographics, lifestyle factors and dietary habits^(18)^. Detailed descriptions of the NOWAC study have previously been published elsewhere^(18)^. The current study used a study sample of 101 316 women aged 41–76, who answered an eight-page FFQ. Questionnaires sent out before 1996 did not include an FFQ; therefore, only women who answered questionnaires between 1996 and 2005 were included. Some of the participants also answered a second questionnaire about 6 years after enrolment (*n* 67 527).

**Fig. 1.**
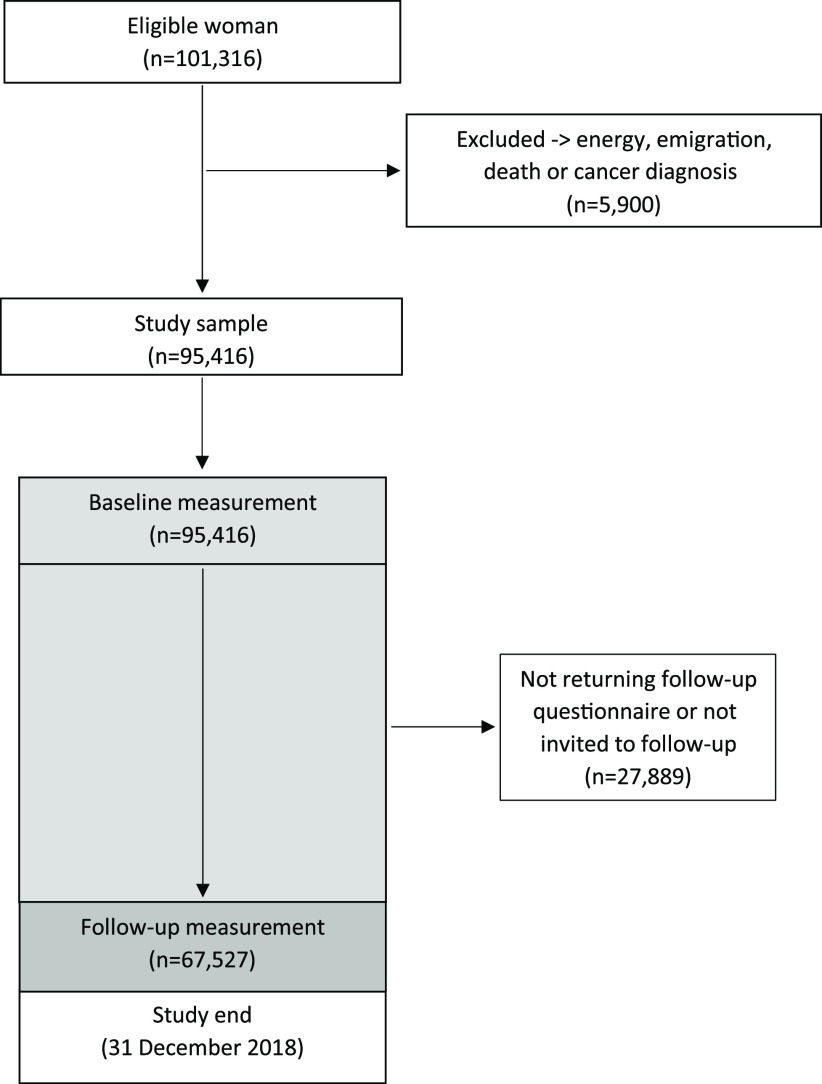
Flow chart of women included in the analysis.

We excluded women who had a current or previous cancer diagnosis at baseline (except for non-melanoma skin cancer) (*n* 4343). Further, we excluded women with no follow-up time (*n* 10), and those with an implausible energy intake (> 3500 kcal, *n* 841 or < 500 kcal, *n* 706) at baseline or follow-up, leaving a total of 95 416 women for the analyses, of which 67 527 had responded to the second FFQ (Fig. 1).

All the women who have participated in NOWAC have given informed consent. The study is approved by the Norwegian Data Protection Authority and Regional Committees for Medical and Health Research Ethics (REK). The women can withdraw whenever they want without giving any explanation for it.

### Assessment of vitamin D intake and other variables

Dietary intake was estimated using a validated FFQ^(20)^. The FFQ used in NOWAC was designed to measure food intake over the past year and adapted to a Norwegian diet with special emphasis on fish consumption. The response options were fixed frequencies, household units, natural units or decilitres for portion sizes. Missing frequency values were treated as no consumption, and missing portion sizes were set to the smallest portion size alternative. The Norwegian Food Composition Database^(21)^ was used to calculate nutrient content in foods, and the Norwegian Weight and Measurement Table ^(22)^ was used to convert the frequencies into grams.

Vitamin D intake was calculated from reported consumption of fatty fish, liquid cod liver oil, eggs and vitamin D-fortified foods like butter/margarine and milk. Estimated fish intake and intake of essential fatty acids in NOWAC have previously been validated^(23)^. Intake of vitamin D was categorised into three groups: low intake [< 10 µg/d], medium intake [10–19 µg/d] and high intake [≥ 20 µg/d].

**Fig. 2.**
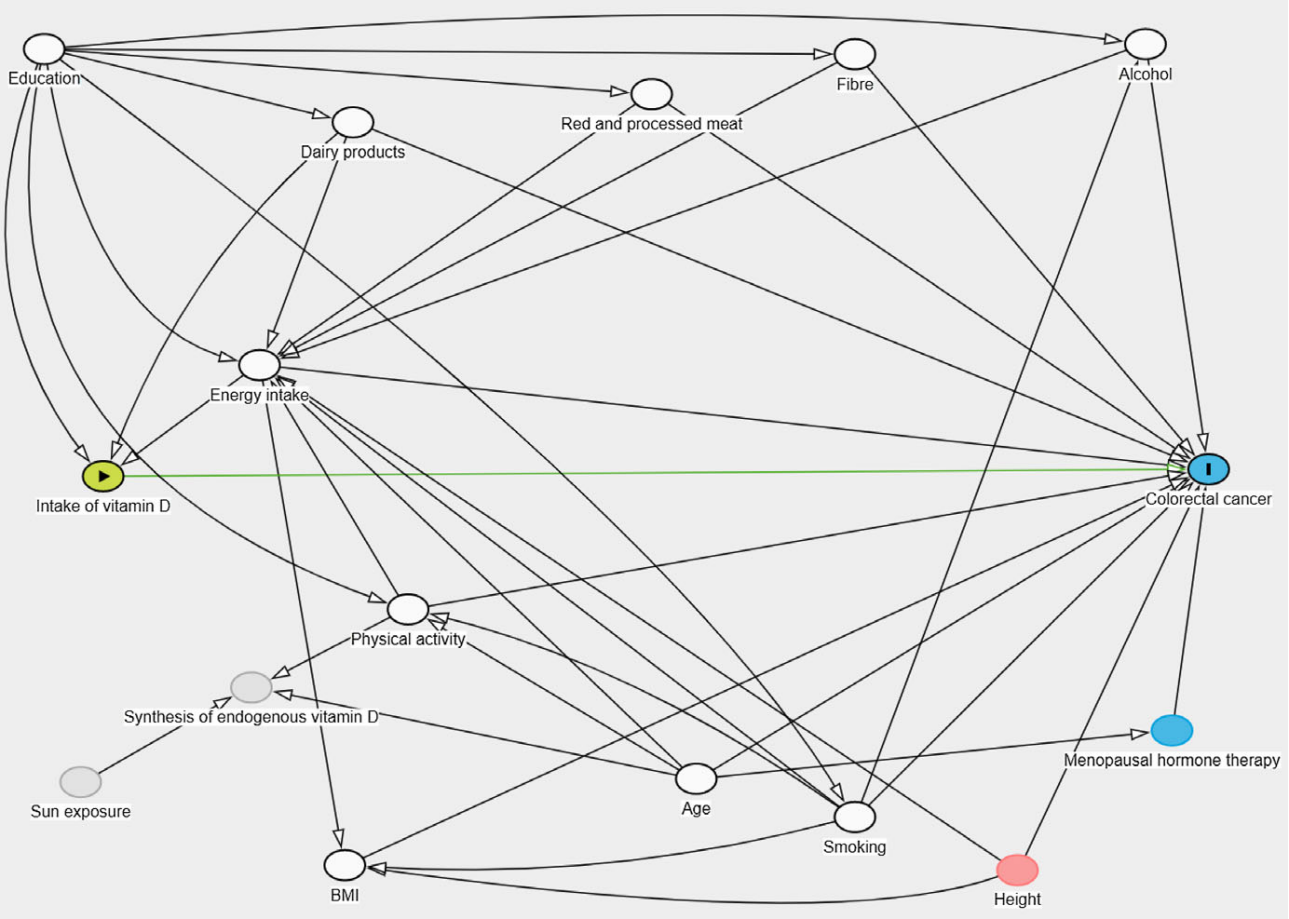
Directed acyclic graph over the association between intake of vitamin D and CRC, green and blue node, respectively. White nodes are identified as confounding factors, and grey nodes are unobserved factors. The red node is a confounding factor on the pathway intake of vitamin D-> energy intake-> height-> colorectal cancer. However, by adjusting for energy intake this pathway will be closed and thus adjustment for height is not necessary. The blue node, menopausal hormone therapy, is an ascendent of the outcome.

Potential confounding factors were *a priori* identified through previous literature and with the use of a directed acyclic graph (Fig. 2)^(24)^. The following potential confounders were included in the analyses: age, smoking, BMI, physical activity, education, and consumption of red meat, processed meat, alcohol, dairy products, and fibre.

Age was retrieved from the linkage to the National Registry in Norway. Information about smoking was based on reported smoking history and smoking status and categorised as never, former or current smoker. BMI (weight/height^2^) was calculated based on validated self-reported weight (kg) and height (metres) and categorised according to WHO’s cut-off values^(25)^: (underweight [BMI < 18·5 kg/m²], normal weight [BMI 20–24·9 kg/m²], overweight [BMI 25–29·9 kg/m²] and obese [BMI ≥ 30 kg/m²]). Physical activity was reported on a scale from 1 to 10, including physical activity at home and at work, as well as exercise and walking, and categorised as: (low [1–4], medium [5–6] and high [7–10]). Education was extracted from the baseline questionnaire and categorised by duration representing compulsory primary school, upper secondary education, and higher education and categorised as (low [< 10 years], medium [10–12 years], and high [> 12 years]). Intake of energy, alcohol and other dietary covariates was estimated from the FFQ, both at baseline and at follow-up. Red meat was calculated by combining the intake of chops, beef and steak. Processed meat was calculated by combining the intake of sausages, meatballs and cold cuts. Dairy products were calculated based on milk and cheese intake. Red meat, processed meat, dairy products and fibre intake were categorised into tertiles based on daily intake. The intake of alcohol was categorised according to the Norwegian Directorate of Health^(26)^: (no intake [0 g/d], requested [< 10 g/] and high [≥ 10 g/d]). Energy intake was coded as a continuous variable and reported in kilocalories (kcal/d)^(27,28)^.

### Outcome

The incident cases of CRC were identified through linkage to the Cancer Registry of Norway. The CRC cases in this study were identified through the International Classification System (ICD)-10 codes C18–20^(29)^ and the ICD-0–3 topography codes C199 and C209^(30)^. We grouped all incident cases of cancer that were located proximally to the splenic flexure as right-sided/proximal colon cancer, while all incident cancer cases that were distal to the splenic flexure were classified as left-sided/distal colon cancer^(31)^. An overview of each intestinal section and the ICD codes included can be seen in Supplementary Table 2.

### Statistical methods

Distribution of covariates at baseline are presented as percentages or median values and 5th–95th percentiles. All women were followed from baseline and until incidence of CRC, emigration, death or end of follow-up (31 December 2018), whichever occurred first. The association between vitamin D intake (each 5 ug/d and categorised) and incidence of CRC, colon cancer, proximal colon cancer, distal colon and rectal cancer was analysed using Cox proportional hazards regression. We used two models (age- and multivariable-adjusted analysis) with age as the timescale. In the multivariable-adjusted analyses, we excluded women with missing information on any of the included covariates. Intake of vitamin D was investigated as a continuous variable (each 5 µg increase in intake per d) and as a categorical variable (low intake: < 10 µg/d, medium intake: 10–19 µg/d and high intake: ≥ 20 µg/d). Low intake was used as the reference category.

The analysis results were reported as hazard ratios (HR) and 95 % CI. A *P*-value < 0·05 was considered significant. The proportional hazards assumption was tested with the Schoenfeld residual test. The statistical analysis was performed using Stata/MP 16·1.

## Results

During follow-up, 1774 women were diagnosed with CRC. Of these, 1228 incident cases of cancer were localised in the colon – 726 in the proximal and 467 in the distal part of the colon. Lastly, 465 of the incident cases of cancer were localised in the rectum (online Supplementary Table 3). The mean age at diagnosis was 56 years.

The women who were diagnosed with cancer had a median follow-up time of about 6 years (range 1–17 years), while those who did not develop cancer had a median follow-up time of 11 years (range 1–17 years).

The median and mean vitamin D intake in the total study sample was 6·0 and 8·9 µg/d at baseline and 6·8 and 9·7 µg/d at follow-up, respectively (online Supplementary Table 3).

At baseline, the median intake of vitamin D in the low, medium and high intake groups was 4·7, 13·9 and 27·9 µg/d, respectively, and 5·3, 13·7 and 28·6 µg/d at follow-up (Table 1). In the total study sample, 72 % (*n* 68 276) had a baseline vitamin D intake < 10 µg/d, 18 % (*n* 17 269) had an intake of 10–19 µg/d and 10 % (*n* 9871) had an intake ≥ 20 µg/d (Table 1). Likewise, 71 % (*n* 1261) of the incident cases of CRC had a vitamin D intake < 10 µg/d, 19 % (*n* 337) had an intake of 10–19 µg/d and 10 % (*n* 176) had an intake ≥ 20 µg/d (Table 1). The mean BMI was 24·0 kg/m^2^ among those in the low intake group of vitamin D, and 25·0 kg/m^2^ for both the medium and high intake group. There were more smokers among those in the low intake group of vitamin D (30 %) compared with those in the medium and high intake groups (28 %). Additionally, more woman reported a low physical activity level in the low intake group of vitamin D (29 %) compared with those in the medium (23 %) and high (20 %) intake groups (Table 1). A lower intake of dairy products was reported in the low intake group of vitamin D (166 g/d) compared with the medium (220 g/d) and high intake group (230 g/d) (Table 1). Lastly, vitamin D intake increased with increasing energy intake. Seventy-one per cent of the woman did not change their vitamin D intake from baseline to follow-up, 14 % reported a lower intake and 15 % reported a higher intake at follow-up (online Supplementary Table 3).

**Table 1.** Selected characteristics of the study sample (*n* 95 416) by vitamin D intake at baseline (1996–2005). The Norwegian Women and Cancer Cohort Study

	Vitamin D intake grouped					
	Low (<10 µg)		Medium (10–19 µg)		High (≥ 20 µg)	
	Median	5th–95th percentile	Median	5th–95th percentile	Median	5th–95th percentile
Number of women at baseline	68 276		17 269		9871	
Number of CRC cases	1261		337		176	
Range of total vitamin D intake at baseline (µg/d)*	4·7	1·6–8·9	13·9	10·3–18·9	27·9	20·9–35·0
Range of total vitamin D intake at follow-up (µg/d)	5·3	2·0–9·2	13·7	10·3–18·9	28·6	20·7–36·2
Age at baseline (years)	51	42–65	52	42–67	53	43–67
BMI at baseline (kg/m²)†	24	19·7–32·4	24	19·6–31·2	24	19·5–30·4
Smoking at baseline (%)‡						
Never	35		38		37	
Former	35		34		35	
Current	30		28		28	
Physical activity at baseline (%)§						
Low	29		23		20	
Medium	43		43		42	
High	28		34		38	
Years of schooling at baseline (%)||						
< 10 years	26		26		27	
10–12 years	35		32		33	
> 12 years	39		42		40	
Intake of dairy products at baseline (g/d)**	166	10·0–600·0	220	14·3–650·0	230	14·3–687·5
Intake of red meat at baseline (g/d)††	13	0–35·4	13	0–35·4	13	0–34·5
Intake of processed meat at baseline (g/d)¶,‡‡	30	4·1–72·9	29	4·1–72·6	28	4·1–70·9
Intake of alcohol at baseline (g/d)	2	0–11·6	2	0–12·4	2	0–12·3
Intake of fibre from foods at baseline (g/d)	20	10·5–31·7	23	12·6–34·6	23	12·7–35·5
Energy intake at baseline (kcal/d)	1559	924·8–2315·9	1793	1121·7–2643·3	1833	1174·3–2699·7

* Vitamin D intake is based on all foods containing vitamin D + liquid cod liver oil.

† *n* 93 250 at baseline.

‡ *n* 94 225 at baseline.

§ *n* 86 708 at baseline.

|| *n* 90 039 at baseline.

¶ *n* 93 967 at baseline.

** Dairy products = milk + cheese.

†† Read meat = steak + chops + beef.

‡‡ Processed meat = sausage + meatballs + cold cuts.

### Colorectal cancer

Each 5 µg increase in vitamin D intake per d was associated with 3 % borderline significant reduced risk for CRC (multivariable-adjusted HR = 0·97; 95 % CI 0·93, 1·01) (Table 2).

**Table 2.** Risk of colorectal cancer, total colon cancer, proximal colon cancer, distal colon and rectal cancer according to vitamin D intake using repeated measurements analyses. The Norwegian Women and Cancer Cohort Study (*n* 95 416)

Vitamin D intake repeated measurements analyses	Age-adjusted analyses				Multivariable analyses*			
	Number of cases	HR	95 % CI	*P*	Number of cases	HR	95 % CI	*P*
Colorectal cancer								
Continuous (5 µg increase)	1774	0·96	0·93, 0·99	0·01	1198	0·97	0·93, 1·01	0·13
Grouped								
Reference value (< 10 µg)	349	1·00			227	1·00		
10–19 µg	1260	0·95	0·84, 1·07	0·36	859	0·96	0·83, 1·12	0·60
≥ 20 µg	165	0·77	0·65, 0·90	0·00	112	0·83	0·68, 1·02	0·07
Total colon cancer								
Continuous (5 µg increase)	1228	0·96	0·93, 1·00	0·04	828	0·96	0·91, 1·01	0·08
Grouped								
Reference value (< 10 µg)	237	1·00			146	1·00		
10–19 µg	870	0·91	0·79, 1·05	0·22	604	0·86	0·72, 1·04	0·12
≥ 20 µg	121	0·80	0·66, 0·96	0·02	78	0·81	0·64, 1·04	0·10
Proximal colon cancer								
Continuous (5 µg increase)	726	0·98	0·94, 1·03	0·36	483	0·95	0·89, 1·01	0·12
Grouped								
Reference value (< 10 µg)	134	1·00			77	1·00		
10–19 µg	513	0·85	0·70, 1·03	0·10	358	0·73	0·57, 0·94	0·02
≥ 20 µg	79	0·86	0·68, 1·09	0·21	48	0·81	0·59, 1·11	0·18
Distal colon cancer								
Continuous (5 µg increase)	467	0·94	0·89, 1·00	0·06	320	0·98	0·91, 1·06	0·62
Grouped								
Reference value (< 10 µg)	94	1·00			62	1·00		
10–19 µg	332	0·99	0·79, 1·25	0·94	229	1·06	0·79, 1·42	0·70
≥ 20 µg	41	0·74	0·53, 1·02	0·07	29	0·87	0·58, 1·29	0·48
Rectal cancer								
Continuous (5 µg increase)	465	0·95	0·89, 1·01	0·10	310	1·00	0·93, 1·08	0·97
Grouped								
Reference value (< 10 µg)	95	1·00			68	1·00		
10–19 µg	333	1·01	0·80, 1·27	0·93	213	1·20	0·91, 1·60	0·21
≥ 20 µg	37	0·67	0·48, 0·94	0·02	29	0·88	0·59, 1·32	0·54

* Multivariable analyses are adjusted for: smoking status, read meat, processed meat, dairy products, alcohol, fibre, BMI, physical activity, education and energy.

Women with a vitamin D intake of 10–19 µg/d compared with the reference category < 10 µg/d had 4 % reduced risk of CRC (multivariable-adjusted HR = 0·96; 95 % CI 0·83, 1·12), although it was non-significant. In contrast, woman with a vitamin D intake of ≥ 20 µg/d, compared with the reference category < 10 µg/d had 17 % borderline significant reduced risk of CRC (multivariable-adjusted HR = 0·83; 95 % CI 0·68, 1·02) (Table 2).

### Colon cancer

Each 5 µg increase in vitamin D intake per d was associated with 4 % borderline significant reduced risk of colon cancer (multivariable-adjusted HR = 0·96; 95 % CI 0·91, 1·01) (Table 2).

Women with a vitamin D intake of 10–19 µg/d compared with the reference category < 10 µg/d had 14 % borderline significant reduced risk of colon cancer (multivariable-adjusted HR = 0·86; 95 % CI 0·72, 1·04) (Table 2). Similarly, vitamin D intake ≥ 20 µg/d compared with the reference category 10 µg/d was associated with 19 % borderline significant reduced risk of colon cancer (multivariable-adjusted HR = 0·81; 95 % CI 0·64, 1·04) (Table 2).

### Proximal colon cancer

Each 5 µg increase in vitamin D intake per d was associated with 5 % borderline significant reduced risk of proximal colon cancer (multivariable-adjusted: HR = 0·95; 95 % CI 0·89, 1·01) (Table 2).

Women with a vitamin D intake of 10–19 µg/d compared with the reference category < 10 µg/d had 27 % lower risk of proximal colon cancer (multivariable-adjusted HR = 0·73; 95 % CI 0·57, 0·94). Although not statistically significant, the multivariable analysis displayed 19 % reduced risk of proximal cancer with a vitamin D intake ≥ 20 µg/d compared with the reference category < 10 µg/d (multivariable-adjusted HR = 0·81; 95 % CI 0·59, 1·11) (Table 2).

### Distal colon cancer

No significant association was observed between a 5-µg increase in vitamin D intake per d and distal colon cancer (multivariable-adjusted HR = 0·98; 95 % CI 0·91, 1·06) (Table 2).

There was no association with distal cancer among the women with a vitamin D intake of 10–19 µg/d compared with the reference category < 10 µg/d (HR = 1·06; 95 % CI 0·79, 1·42) (Table 2). However, although not significant, the women with a vitamin D intake of ≥ 20 µg/d compared with the reference category < 10 µg/d had a 13 % reduced risk of distal cancer (multivariable-adjusted HR = 0·87; 95 % CI 0·58, 1·29) (Table 2).

### Rectal cancer

No significant association was observed between a 5 µg increase in vitamin D intake per d and rectal cancer (multivariable-adjusted HR = 1·00; 95 % CI 0·93, 1·08) (Table 2).

Compared with the reference category < 10 ug/d, woman with a vitamin D intake of 10–19 µg/d or an intake of ≥ 20 µg/d displayed no association with rectal cancer (multivariable-adjusted HR = 1·20; 95 % CI 0·91, 1·60) (multivariable-adjusted HR = 0·88; 95 % CI 0·59, 1·32), respectively (Table 2).

## Discussion

In this study, we found that medium vitamin D intake (10–19 µg), compared with low intake (< 10 µg/d), significantly decreased the risk of proximal colon cancer by 27 % (multivariable-adjusted). In addition, high (≥ 20 ug/d) compared with low intake of vitamin D (< 10 µg/d) was associated with a 17 % borderline significant reduced risk of CRC. Lastly, each 5 µg increase in vitamin D intake per d was associated with 3 and 4 % borderline significant reduced risk of CRC and colon cancer, respectively. No association was found between vitamin D intake and distal colon or rectal cancer. This study’s results indicate that vitamin D intake is associated with risk of colon cancer at some anatomical subsites.

To the best of our knowledge, there are few other studies that have examined intake of vitamin D and risk of CRC in the same way as we did in the present study: by cancer in anatomical subsites of the colon and the use of repeated measurements. In our study, 70 % of the CRC cases had cancer localised in the colon, of which approximately 60 % were categorised as proximal and 40 % as distal cancer, and 30 % had cancer localised in the rectum.

There are indications that elderly women have a higher risk of proximal colon cancer, while distal colon cancer seems to be more common among men and in younger age groups^(32,33)^. McCullough et al.^(12)^ found a stronger association between vitamin D intake and distal colon cancer compared with proximal colon cancer in a sample that included men^(12)^. However, they did not present analyses on subsites among woman due to small numbers of cases^(12)^. It is plausible that vitamin D has different prognostic value depending on the molecular cause of the cancer as there is evidence for proximal colon cancer and distal colon cancer being driven to various degrees by different molecular causes^(32–35)^. Proximal colon cancer appears to be driven mainly by microsatellite instability and CpG island methylator phenotype^(32–35)^, while distal colon cancer appears to be driven by chromosomal instability^(33–35)^. In addition, it is believed that mutations in different genes dominate in the different subsites, and that several different signalling pathways, proteins and genes are involved^(32)^. Furthermore, it seems that fluoracil-based cytotoxic drugs have different sensitivities in the different subsites^(33)^.

In our study, no association was found between vitamin D intake and rectal cancer. Similar results have been found in several other studies^(9,10,12)^. In contrast, Martinez et al.^(6)^ found that for each 6·25 µg increase in intake of vitamin D from food per d, the risk of rectal cancer was reduced by 55 % (HR = 0·45;95 % CI 0·25, 0·83)^(6)^. However, the association weakened when they investigated the total intake of vitamin D (foods and supplements) and when they included three repeated measurements of vitamin D intake^(6)^. That said, there are already indications that development of rectal cancer is related to other causal factors than for colon cancer. For example, physical activity is associated with a reduced risk of colon cancer, but not for rectal cancer, and vitamin C intake is currently only associated with colon cancer^(2)^. Thus, it is therefore not unlikely that vitamin D also can be differently associated with each subsite.

Results from studies investigating the association between vitamin D and CRC are inconsistent^(6–15,36)^. The biological mechanism of a potential relation between vitamin D intake and risk of proximal colon cancer requires further investigation, in addition to being verified by other studies. That said, inflammation seems to be closely linked to the development of CRC^(37)^. This can be reflected by the increased risk of CRC amongst patients with irritable bowel diseases, and the decreased risk amongst long time users of the non-steroidal anti-inflammatory drug aspirin^(3)^. Vitamin D is believed to have immunomodulatory roles and may reduce pro-inflammatory- and increase anti-inflammatory cytokines which also is involved in CRC^(37)^. It has been proposed that vitamin D may influence the progression of CRC by modulating inflammatory markers, and that future studies should explore this further^(37)^. The causes to the observed relationship between vitamin D intake and proximal part of colon remain unknown.

Research on vitamin D and health has several methodological challenges. There has been some controversy around defining an optimal level of circulating vitamin D. Thus, previous studies have used various ways of defining sufficient or optimal levels of circulating vitamin D. Different cut-offs for intakes can also be considered as a methodological challenge that makes it difficult to compare the results. In this study, we used intakes as exposure and grouped the intake in line with the Nordic Nutrition Recommendations for optimal bone mineralisation^(4)^, to assess if the recommendation could also be associated with reduced risk of CRC and/or its respective subsites. In comparison, other studies that group the intake of vitamin D into, for example, tertiles or quartiles based on the population intake within the study sample risk having too narrow exposure range. Thus, sufficiently high serum values according to recommendations will not be obtained.

Due to the contribution of UVB light on vitamin D status, it is likely that dietary intake of vitamin D has different associations on cancer risk in diverse populations^(38,39)^. The endogenous production of vitamin D will be lower for populations living in geographical areas with low exposure to sunlight. Intake through foods will therefore be of greater importance to maintaining adequate serum levels for the women in this study, as endogenous production will be low for large parts of the year. A stated challenge, especially for clinical trials on vitamin D, is believed to be the lack of a true control group, as vitamin D sufficient people are included and therefore no beneficial association can be observed^(40)^.

The main strengths of our study include its prospective design and long-term follow-up. The women included were randomly selected through the National Registry in Norway, in which it is also possible to follow them almost completely to end points (diagnosis, death or emigration). In addition, previous evaluation of the Norwegian Cancer Registry showed that it contains nearly complete information about CRC cases, most of which are morphologically verified^(41)^. Except for a somewhat higher level of education, no major differences have been found between respondents and non-responders in NOWAC. Therefore, we have reasons to believe that the study has good external validity^(42)^.

The intake of vitamin D was measured before the time of diagnosis and estimated with the use of two repeated measurements. Even at 6-year intervals, 71 % reported the same intake of vitamin D. Validation on fish intake, one of the main sources of vitamin D, showed a good correlation with serum phospholipids^(23)^, and the FFQ in NOWAC has previously been validated^(20)^.

There are also several limitations in our study. No data were used for the estimation of UVB exposure. Thus, for many, the main source to vitamin D was not accounted for. Further, there is some evidence for the role of Ca as a risk factor for CRC^(43)^. In our analysis, Ca intake was not included; however, we adjusted for dairy products as a proxy for intake of Ca. FFQ captures the general intake from the previous year and is thus subject to the participant’s ability to recall their diet. The answers given can be influenced by how the diet is at the time they respond to the FFQ and thus give rise to incorrect information. Also, the subjective measurements may give rise to under-reporting of unhealthy foods and over-reporting of healthy foods. Furthermore, there may be some underestimation of the intake, as no dietary supplements other than cod liver oil have been accounted for. Even though we carefully selected adjustment factors, measurement errors are likely, so residual confounding cannot be ruled out. The covariates included in the directed acyclic graph were based on previously published literature on vitamin D and CRC. However, as described previously, there could be different causal factors associated with each anatomical subsite, and future studies may take this into account when adjusting for confounding factors. However, this hypothesis is not well established, and we therefore did not find it reasonable to make different directed acyclic graphs for each anatomical subsite.

Our study indicates that vitamin D intake is associated with reduced risk of colon cancer at some anatomical subsites, and future studies should therefore try to replicate our findings. If not, important findings may not be discovered, as vitamin D seems to have different associations with different subsites of the colon. In addition, the final vitamin D status can be affected by genetics, age, pigment, sex, metabolism, diseases, medications and sun exposure^(44)^, which means that it should be considered measuring serum 25(OH)D in parts of the cohort to see if the intake reflects the actual vitamin D status.

### Conclusion

Vitamin D intake was associated with reduced risk of proximal colon cancer. No association was found with distal colon or rectal cancer. This study indicates that vitamin D may be differently associated with subsites of the colon. As almost 71 % of the participants had intakes below the current vitamin D recommendation, the CRC cancer preventive potential of increasing vitamin D status in the population may be high, and findings from this study should be further verified.

## Supplementary material

For supplementary material accompanying this paper visit https://doi.org/10.1017/S0007114523000077. click here to view supplementary material
