# Supplementary material for: Pre-diagnostic intake of vitamin D and incidence of colorectal cancer by anatomical subsites: the Norwegian Women and Cancer Cohort Study (NOWAC)
Source: Br J Nutr. 2023 Jan 9;130(6):1047–55. doi: 10.1017/S0007114523000077 (PMC10442793; doi:10.1017/S0007114523000077)

**Supplementary**

**Supplementary Table 1** Intestinal sections and the ICD-codes included in the survival analysis


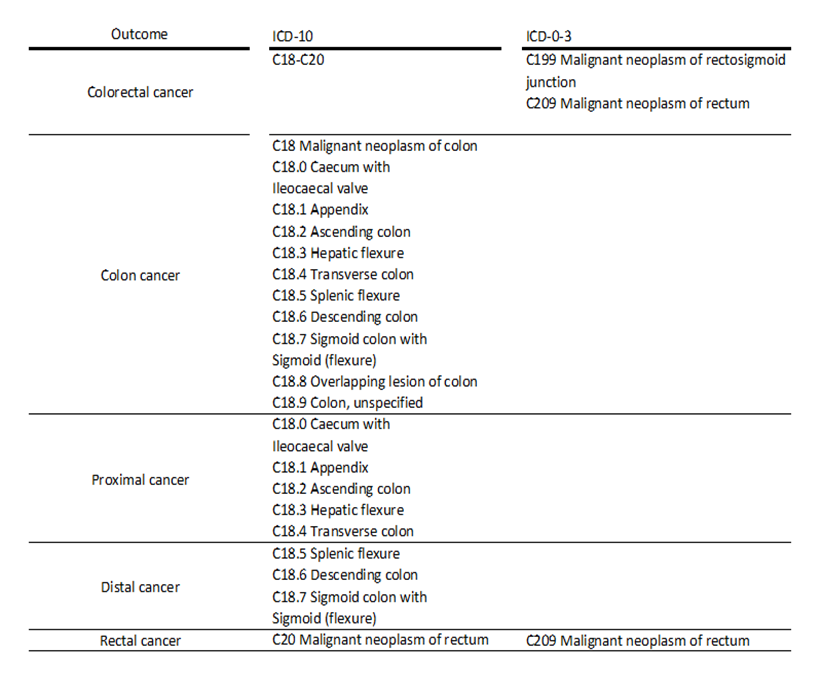


**Supplementary Table 2** Selected characteristics of the study sample (n=95416) by vitamin D intake and colorectal cancer, colon cancer, proximal colon cancer, distal colon and rectal cancer at baseline (1996-2005). The Norwegian Woman and Cancer Cohort Study.


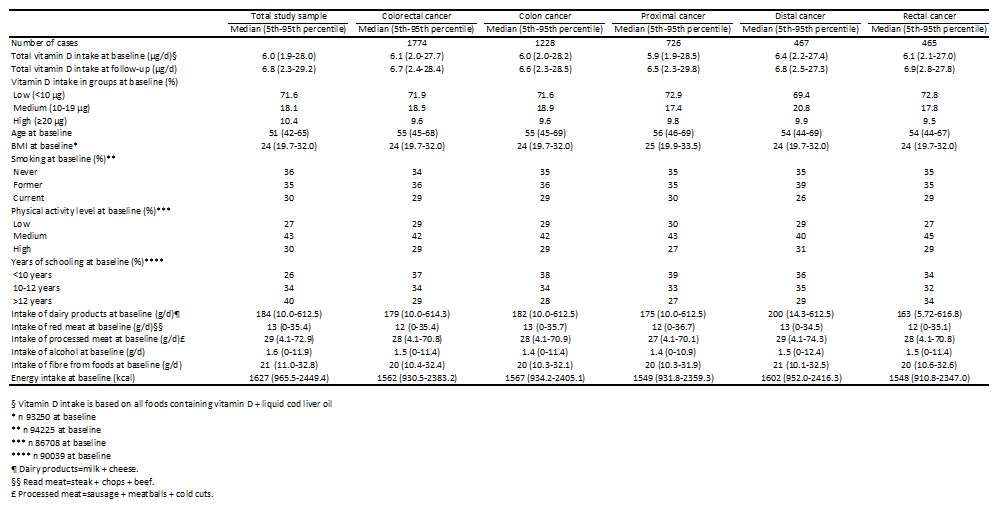


**Supplementary Table 3** Percentage of vitamin D intake at baseline and follow-up (n 67527). The Norwegian Woman and Cancer Cohort Study (NOWAC).


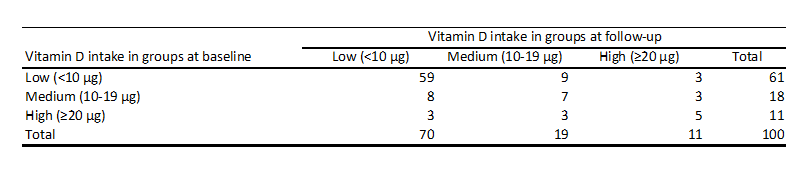

Supplement: Supplementary file 1 [file S0007114523000077sup001.docx]
